# Effects of human mesenchymal stem cells on ER-positive human breast carcinoma cells mediated through ER-SDF-1/CXCR4 crosstalk

**DOI:** 10.1186/1476-4598-9-295

**Published:** 2010-11-18

**Authors:** Lyndsay V Rhodes, James W Antoon, Shannon E Muir, Steven Elliott, Barbara S Beckman, Matthew E Burow

**Affiliations:** 1 Department of Medicine, Section of Haematology and Medical Oncology, Tulane University Health Science Centre, New Orleans, Louisiana, USA; 2 Department of Pharmacology, Tulane University Health Science Centre, New Orleans, Louisiana, USA; 3 Centre for Bioenvironmental Research, Tulane University Health Science Centre, New Orleans, Louisiana, USA; 4 Tulane Cancer Centre, Tulane University Health Science Centre, New Orleans, Louisiana, USA

## Abstract

**Background:**

Adult human mesenchymal stem cells (hMSC) have been shown to home to sites of carcinoma and affect biological processes, including tumour growth and metastasis. Previous findings have been conflicting and a clear understanding of the effects of hMSCs on cancer remains to be established. Therefore, we set out to investigate the impact of hMSCs on the oestrogen receptor positive, hormone-dependent breast carcinoma cell line MCF-7.

**Results:**

In this study, we show the effects of hMSCs on cancer cells are mediated through a secreted factor(s) which are enhanced by cancer cell-hMSC contact/communication. In addition to enhanced proliferation when in co-culture with hMSCs, MCF-7 cells were found to have increased migration potential *in vitro*. Inhibition of ER signalling by the pure anti-oestrogen ICI 182,780 decreased the effect of hMSCs on MCF-7 cell proliferation and migration supporting a role for ER signalling in the hMSC/MCF-7 cell interaction. Additionally, hMSCs have been shown to secrete a wide variety of growth factors and chemokines including stromal cell-derived factor-1 (SDF-1). This coupled with the knowledge that SDF-1 is an ER-mediated gene linked with hormone-independence and metastasis led to the investigation of the SDF-1/CXCR4 signalling axis in hMSC-MCF-7 cell interaction. Experiments revealed an increase in SDF-1 gene expression both *in vivo *and *in vitro *when MCF-7 cells were cultured with hMSCs. SDF-1 treatment of MCF-7 cells alone increased proliferation to just below that seen with hMSC co-culture. Additionally, blocking SDF-1 signalling using a CXCR4-specific inhibitor decreased hMSC induced proliferation and migration of MCF-7. However, the combined treatment of ICI and AMD3100 reduced MCF-7 cell proliferation and migration below control levels, indicating targeting both the ER and CXCR4 pathways is effective in decreasing the hMSCs induction of MCF-7 cell proliferation and migration.

**Conclusions:**

The sum of these data reveals the relationship between tumour microenvironment and tumour growth and progression. Better understanding of the mechanisms involved in this tumour stroma cell interaction may provide novel targets for the development of treatment strategies for oestrogen receptor positive, hormone-independent, and endocrine-resistant breast carcinoma.

## Background

Oestrogen receptor-α (ER) status is one of the most widely used prognostic markers of breast carcinoma as it is required for 17β-oestradiol (oestrogen) action, and it has long been known that oestrogen has the ability to promote breast tumour formation and proliferation [1,2]. By blocking oestrogen signalling through the removal of endogenous oestrogen, inhibiting binding of oestrogen to its receptor or blocking ER signalling, the tumour promoting effects of oestrogen can be reversed [2-6]. These effects have been the foundation for the use of targeted therapies such as the anti-hormone therapies tamoxifen and fulvestrant (ICI 182,780) and aromatase inhibitors. Although endocrine therapy holds great promise in the treatment of hormone-dependent cancer, as many as 50% of patients with ER-positive breast carcinoma do not respond to treatment, exhibiting *de novo *resistance to therapy. Furthermore, many patients who initially respond well to treatment will develop tumours which progress to a resistant phenotype [7]. Resistance typically develops through sequential phenotypes from total oestrogen dependence, to hormone independence while retaining oestrogen sensitivity, to complete hormone independence and endocrine therapy resistance [7,8]. Though decreased ER expression is associated with cancer progression many patients advance to hormone independence and/or endocrine therapy resistance while retaining ER positivity [9]. The progression to hormone independence and endocrine therapy resistance are hallmark signs of progressive carcinoma [10,11]. Currently, all endocrine treatments approved for clinical use ultimately result in resistance, demonstrating the ability of carcinoma cells to adapt by altering cellular signalling [12-15].

In recent years, the tumour microenvironment has gained appreciation as an active participant in the processes of tumourigenesis and metastasis as well as in the progression to hormone independence and endocrine therapy resistance [16-18]. The interaction between tumour cells and tumour stroma or microenvironment has been described as a "two-way street" due to the ability of tumour cells to influence the stroma via tissue remodeling and gene expression and vice versa [19-21]. Tumour cells provide signals that stimulate *de novo *formation of basement membrane (BM) and extra-cellular matrix (ECM) in order to provide stromal support to the growing tumour [22,23]. The host response to the establishment of tumour stroma closely mimics that of wound healing and scar development [24] leading not only to modified secreted proteins from tumour cells and stroma (direct action), but also the recruitment of other supporting cell types (indirect action) such as endothelial progenitor cells [25], and mesenchymal stem cells [26-28].

Human mesenchymal stem cells (hMSC) are multipotent progenitor cells that contribute to tissue repair and wound healing [29]. These cells possess the ability to self-renew while retaining the ability to differentiate into cell types of mesenchymal origin including osteoblasts, chondrocytes, and adipocytes [30-33]. Since Paget's original report of the "Seed and Soil" theory in 1889, it has been known that breast cancer cells preferentially metastasize to specific sites, one of which is the bone marrow [34]. hMSCs have been implicated in the interaction of breast cancer cells within the bone marrow via direct contact as well as by secreted factors [35,36]. In addition, hMSCs have the ability to preferentially home to tumour sites, endogenously or when injected systemically, and contribute to the dynamic tumour stroma [37-39].

It has been suggested that hMSCs home to tumour cells and surround the tumours without infiltrating them, indicating that any effects of the hMSCs results from stromal factors and paracrine signaling [40]. hMSCs secrete high levels of cytokines, chemokines, and growth factors basally; yet these secretion profiles can be changed depending on the culture conditions or microenvironment [41]. Others have shown that tumour associated stromal cells contribute to primary tumour growth *in vivo *via chemokine paracrine signaling [42]. One such chemokine is stromal cell-derived factor-1 (SDF-1) along with its receptor CXCR4. hMSCs are known to secrete SDF-1 under normal conditions, and the SDF-1/CXCR4 axis is an important mediator of hMSCs chemotaxis [43] and primary breast tumourigenesis [44]. Previously we have demonstrated hMSCs as having the ability to promote hormone independent growth *in vivo *in the naturally hormone-dependent breast carcinoma cell line MCF-7 [45]. This data, along with the fact that hormone-independent breast carcinomas are associated with a metastatic phenotype, and that SDF-1 is a known ER regulated gene, provide a link between hormone and chemokine signalling in the progression of breast carcinoma cells [10,46,47].

Previous studies have produced conflicting findings as to the effects of hMSCs at the tumour site, yet interest in harnessing the natural homing capabilities for the development of targeted cancer therapy has increased. Though this is an exciting treatment option, proceeding without further knowledge of the effects that hMSCs naturally exert on carcinoma cells could lead to unforeseen and unwanted side effects. In the present study we examined the effects of hMSCs on the proliferative and metastatic potential of the ER-positive, hormone-dependent breast carcinoma cell line MCF-7.

## Results

### hMSCs enhance proliferation of MCF-7 breast cancer cells *in vitro*

**Figure 1:**
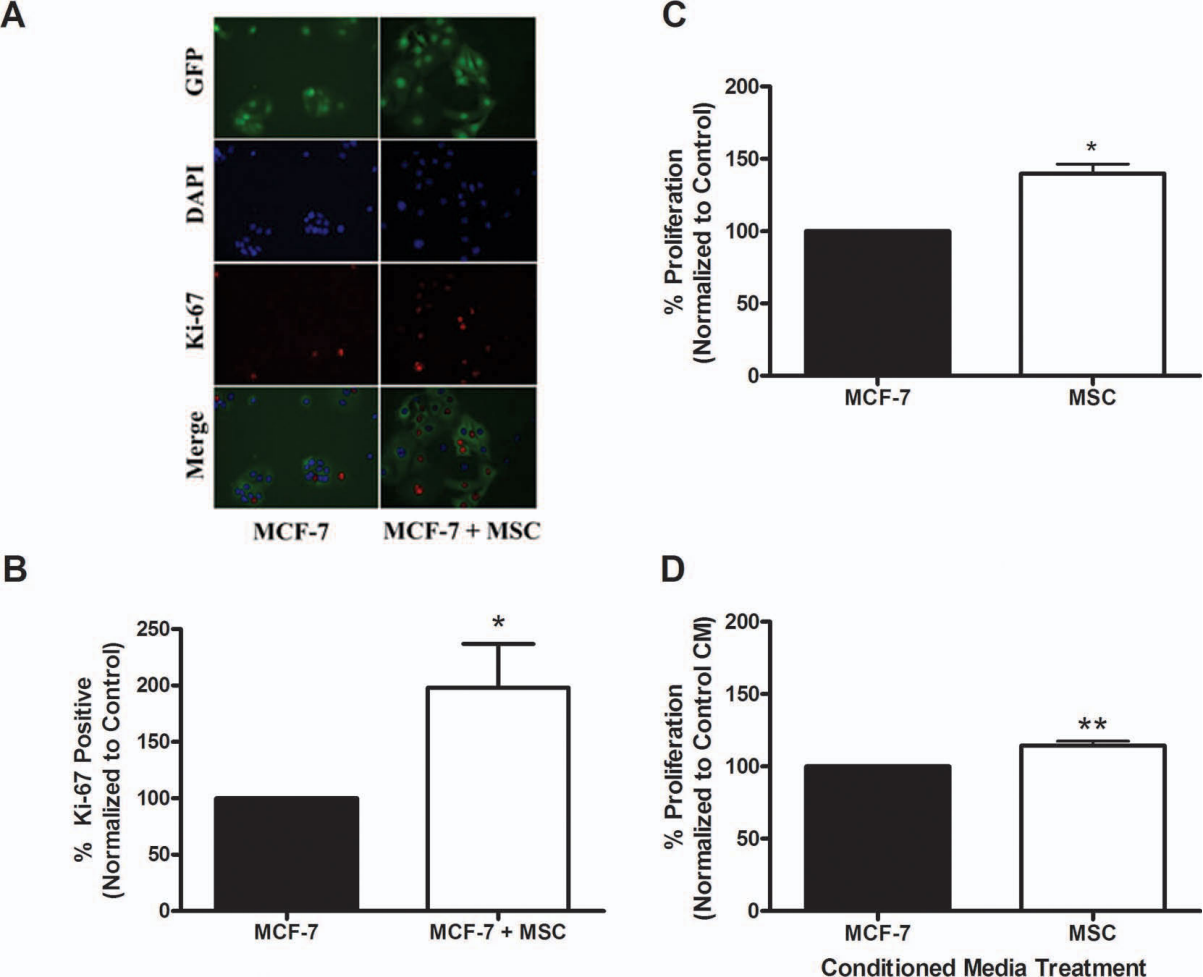
**hMSCs enhance MCF-7 cell proliferation**. (A) MCF-7-GFP cells cultured with or without hMSCs over 72 hours. Cells were fixed and stained with anti-Ki-67 (red) and nuclei were counter stained with DAPI (blue). Representative images of MCF-7 cells cultured alone (left) or with hMSCs (right) at 400×. (B) Quantification of GFP cells positive for Ki-67 staining from 10 fields of view per treatment. Data is represented as percent positive MCF-7 cells as compared to total number of MCF-7 cells normalized to MCF-7 control group. (C) MCF-7 cells were seeded in the lower chamber of a 24-well plate and MCF-7 cells (control) or hMSCs were seeded on the upper transwell insert. After 7 days of culture, inserts were removed and MTT was added to each well. After 4 hours, cells were solubilized and absorbance read. (D) MCF-7 cells were treated with MCF-7 (control) or hMSC conditioned media for 24 hours. After 4 hours of MTT treatment, cells were solubilized and absorbance read. All data are represented as percent proliferation normalized to control treated cells ± SEM, (* p < 0.05, *** p < 0.001).

The effects of hMSCs on primary breast carcinoma have been examined in several studies; however, the resulting data have been conflicting. The effects of hMSCs on MCF-7 cell proliferation were tested under various conditions to determine if secreted factors were involved or if direct cell contact was necessary. Direct co-culture assays were first used to test the effect of hMSCs on MCF-7 cell growth in culture. MCF-7 cells stably transfected with green fluorescence protein (GFP) were utilized in these experiments to delineate the two cell populations (MCF-7 versus hMSC). Immunofluorescence images of Ki-67 staining reveal a visible increase in proliferation in MCF-7 cells cultured with hMSCs after 72 hours of direct co-culture (Figure 1A). Quantification revealed a near doubling in the number of proliferating MCF-7 cells (198.3 ± 38.77%, p < 0.05) when cultured with hMSC compared to MCF-7 cells cultured alone (Figure 1B). hMSCs showed no change in proliferation under these culture conditions (data not shown).

In addition to the direct co-culture assay, transwell proliferation assays were performed to determine if cell-to-cell contact was necessary for enhanced proliferation of MCF-7 cells by hMSCs. MCF-7 cells were plated in the lower wells of a 24-well plate Boyden chamber system under normal culture conditions. Transwell inserts containing either MCF-7 cells (control) or hMSCs were placed over each well. After 7 days, proliferation of the MCF-7 cells in the lower wells was determined by MTT assay. MCF-7 cells grown in the presence of hMSCs show a 39.9 ± 6.537% (p < 0.05) increase in proliferation compared to control (Figure 1C). The results reveal that hMSCs significantly enhance MCF-7 cell proliferation in the absence of direct cell-to-cell contact, though the effect is not as robust as that observed in direct co-culture.

Induction of proliferation in the absence of cell-contact suggests that the effects of hMSC on MCF-7 cells are mediated by a secreted factor(s) able to cross the transwell membrane. To determine if hMSC secreted factors are responsible for increases in MCF-7 cell proliferation, conditioned media experiments were performed. MTT proliferation assays of MCF-7 cells cultured in MCF-7 (control) or hMSC conditioned media (CM) for 48 hours revealed that treatment of MCF-7 cells with hMSC CM resulted in a 14.34 ± 3.09% (p < 0.01) increase in MCF-7 cell proliferation compared to MCF-7 control CM (Figure 1D). Although significant, the effect of hMSC CM treatment on MCF-7 cell proliferation was sufficiently reduced compared to transwell experiments (p < 0.01), indicating that secreted factors may differ between naïve hMSCs and those "activated" in the presence of MCF-7 cells. Differences in proliferative effects between the transwell and CM may also be due to the finite amounts of secreted factors available in CM compared to transwell assays where hMSCs continually secrete factors. However, varying the concentration of CM and/or replenishing cultures with fresh CM did not significantly alter the effect (data not shown).

### hMSCs enhance breast carcinoma cell migration *in vitro*

**Figure 2:**
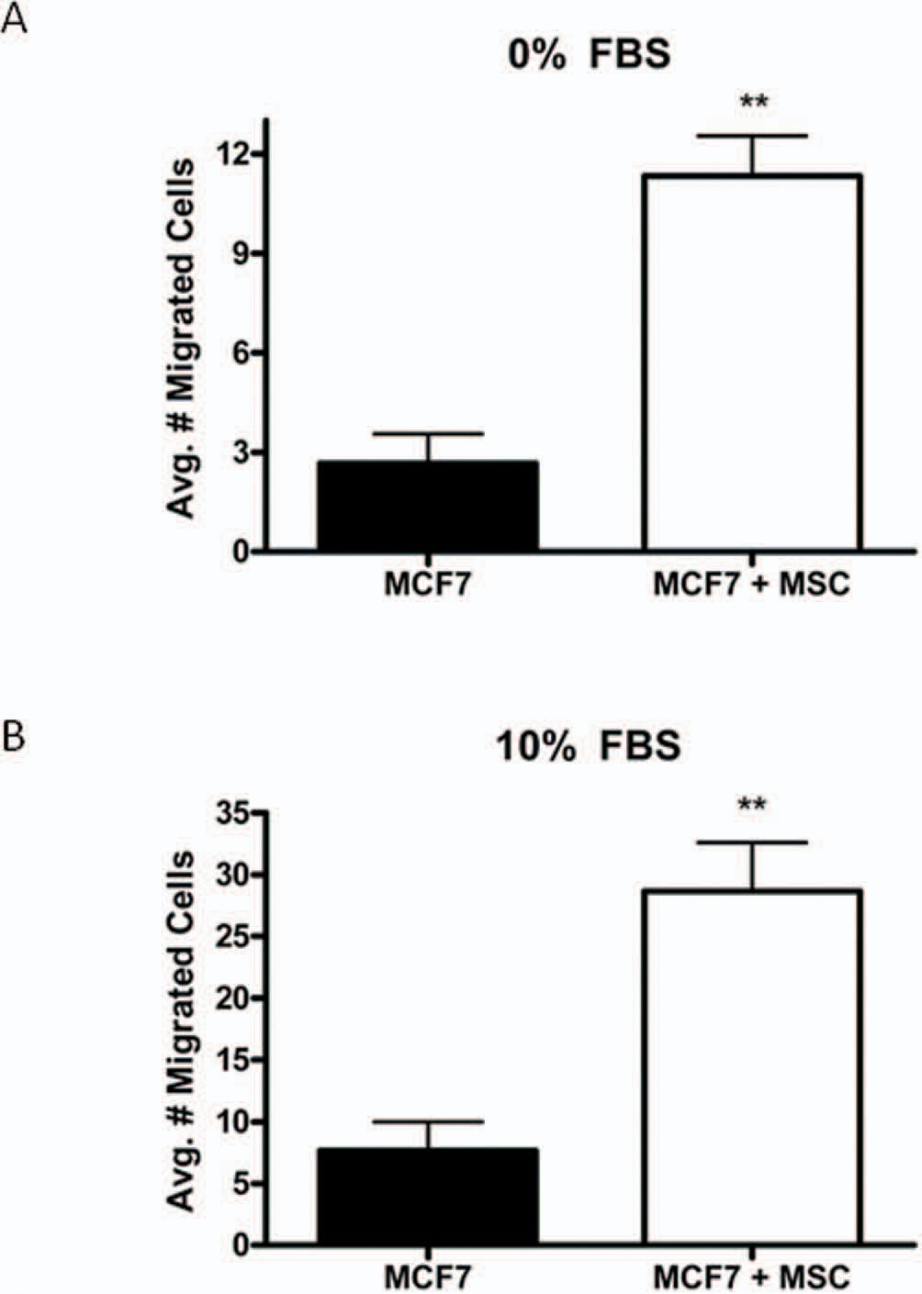
**hMSCs increase migration of MCF-7 cells *in vitro***. MCF-7-GFP cells alone or in combination with hMSCs (1:1) were seeded in the upper chamber of a transwell system and treated with DMSO. Lower wells contained either (A) serum-free culture media (0%) or (B) culture media supplemented with 10% FBS (10%). After 48 hours cells were fixed and the number of GFP-positive migrated cells counted. Bars represent average number of migrated cell per condition ± SEM, (** p < 0.01).

Recent reports indicate that hMSCs increase the metastatic potential of cancer cells [48,49]. In this study we set out to determine if hMSCs affect the migration potential of the normally non-metastatic MCF-7 cells. MCF-7-GFP cells were again utilized to delineate MCF-7 cells from hMSC when in direct culture. We initially tested the ability of hMSCs to increase the basal motility of MCF-7 cells. MCF-7 cells were plated alone or in combination with hMSCs (1:1) in the upper chamber of a transwell culture system and phenol red-free culture media without FBS (0%) in the lower wells. MCF-7 cell migration after 48 hours was increased approximately 4-fold from 2.67 ± 0.88 cells per well when cultured alone (control) to 11.33 ± 1.2 cells per well when cultured directly with hMSCs (Figure 2A, p < 0.01). The effect of hMSCs on stimulated chemotaxis was next examined using 10% FBS as a chemoattractant in the lower chamber. MCF-7 cells cultured in the presence of hMSC demonstrated an increase in migration of 4-fold (28.67 ± 3.93 cells per well) compared to MCF-7 cells cultured alone (7.67 ± 2.33 cells per well) over 48 hours (Figure 2B, p < 0.01). A slight, but insignificant increase in the number of migrated MCF-7 cells (control) was observed (7.67 ± 2.33 cells) as compared to the control conditions (2.67 ± 0.88 cells) demonstrating the very low inherent motility potential of MCF-7 cells; while hMSC induced migration was significantly enhanced in the presence of a chemoattractant from 11.33 ± 1.2 cells to 28.67 ± 3.93 cells per well (p < 0.5; data not shown).

### Effects of ER signaling on hMSC stimulated MCF-7 proliferation and migration

**Figure 3:**
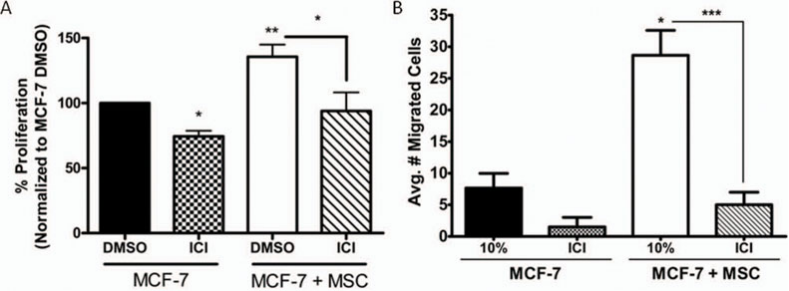
**Inhibition of ER signaling decreases hMSC stimulated MCF-7 proliferation and migration *in vitro***. (A) MCF-7 cells were seeded in the lower chamber of a 24-well plate with MCF-7 cells or hMSCs were seeded in the upper well. Upper and lower chambers were treated with either DMSO (control) or ICI (100 nM). After 7 days of culture, inserts were removed and MTT was added to each well. Cells were solubilized and absorbance read. All data are represented as normalized percent proliferation compared to MCF-7 + DMSO (control) treated cells ± SEM, (* p < 0.05, ** p < 0.01). (B) MCF-7-GFP cells alone or in combination with hMSCs (1:1) were seeded in the upper chamber of a transwell system and lower wells contained culture media supplemented with 10% FBS (10%). Upper and lower chambers were treated with either DMSO (control) or ICI (100 nM). After 48 hours cells were fixed and the number of GFP positive migrated cells counted. Bars represent average number of migrated cell per condition ± SEM, (* p < 0.05, *** p < 0.001).

Building upon our previous observation of a hormone-independent phenotype in MCF-7 cells induced by hMSCs *in vivo *as well as increased progesterone receptor (PgR) expression of MCF-7 + hMSC derived tumours [45,50], we examined the involvement of ER signalling in the hMSC-MCF-7 cell interaction. *In vitro *conditioned media experiments confirmed enhanced expression for the ER-regulated gene PgR in MCF-7 cells grown in the presence of hMSC conditioned media for 24 hours (data not shown). The pure anti-oestrogen ICI 182,780 (ICI) was used in transwell proliferation assays to determine if blocking ER-mediated signalling could inhibit the effect of hMSCs on MCF-7 cell growth. Figure 3A illustrates that inhibition of ER results in the expected decrease of control MCF-7 cell proliferation (from 100% to 74.45 ± 4.2%, p < 0.05), while also inhibiting hMSC stimulated growth of MCF-7 cells approximately 31% (from 135.64 ± 18.93% to 93.88 ± 14.37%), though not significantly. ICI treatment of hMSC-MCF-7 cultures did not result in significant changes from MCF-7 control or MCF-7 + ICI treatment conditions.

We next examined the role of ER in the enhanced migratory response of MCF-7 cells in the presence of hMSCs using the transwell migration assay. After 48 hours, the number of migrated MCF-7 cells was counted and compared to control migration. Inhibition of the ER with ICI resulted in a decrease in the migration of MCF-7 cells cultured alone (from 7.67 ± 2.33 cells to 1.5 ± 1.5 cells). These results were not statistically significant due to very low numbers of basal migration (Figure 3B). hMSC co-culture-induced migration of MCF-7 cells was inhibited with ICI treatment (5 ± 2 cells, p < 0.001), but not significantly below that of control MCF-7 migratory levels (7.67 ± 2.33 cells). These results verify a role for ER activity in hMSC-MCF-7 interaction, but due to the incomplete inhibition of the effects of hMSCs on MCF-7 cells with ICI treatment also suggests that other pathways may be involved in MCF-7 cell response to hMSCs.

### SDF-1/CXCR4 signalling involvement in hMSC mediated effects on MCF-7 cells

**Figure 4:**
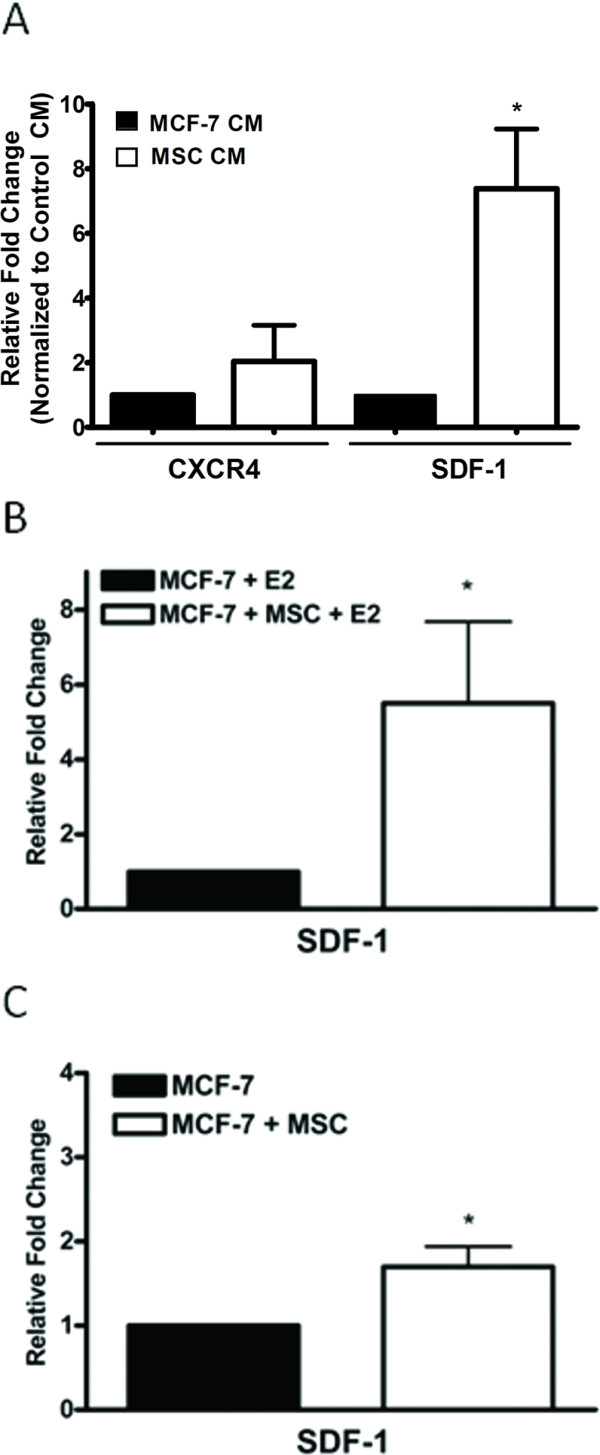
**hMSCs enhance SDF-1 gene transcription *in vivo *and *in vitro***. (A) MCF-7 cells were treated with conditioned media from MCF-7 cells (control) or hMSCs for 24 hours. Cells were then harvested for PCR analysis of CXCR4 and SDF-1. (B) Endpoint oestrogen and matrigel tumours were harvested at day 36 post cell injection and gene expression analyzed by qPCR. (C) Endpoint matrigel tumours grown in the absence of oestrogen were harvested at day 44 and gene expression analyzed by qPCR. Data represent fold change relative to control as normalized to internal β-actin ± SEM, (*, p < 0.05).

Our observations of enhanced ER signalling in the MCF-7hMSC interactions both *in vitro *and *in vivo *supports recent evidence linking ER and CXCR4 chemokine signalling in the progression to hormone independence [47]. Given that hMSCs have been shown to produce copious amounts of stromal-cell derived factor-1 (SDF-1) [51], we next explored the possibility of enhanced SDF-1/CXCR4 signalling in our model system. MCF-7 cells in culture were treated with CM from MCF-7 cells (control) or hMSCs for 48 hours and gene expression analyzed by qPCR. Results show no significant change in CXCR4 expression levels (2.04 ± 1.12 fold), but a 7.39 ± 1.84 fold increase (p < 0.05) in SDF-1 gene expression levels (Figure 4A).

To confirm *in vitro *gene expression data, matrigel tumour samples from our previous primary tumour study [45] were subjected to qPCR analysis for SDF-1 gene expression. Tumours grown in the presence of hMSCs and E2 showed a significant increase in of SDF-1 gene expression (5.5 ± 2.18 fold, p < 0.05) when compared to MCF-7 + E2 control tumours (Figure 4B). Similarly, matrigel only tumours demonstrated increased SDF-1 gene expression in tumours from the MCF-7/hMSC group (1.7 ± 0.24 fold, p < 0.05) (Figure 4C). These data suggest possible involvement of SDF-1/CXCR4 signalling in the hMSC effects on MCF-7 cell biology.

**Figure 5:**
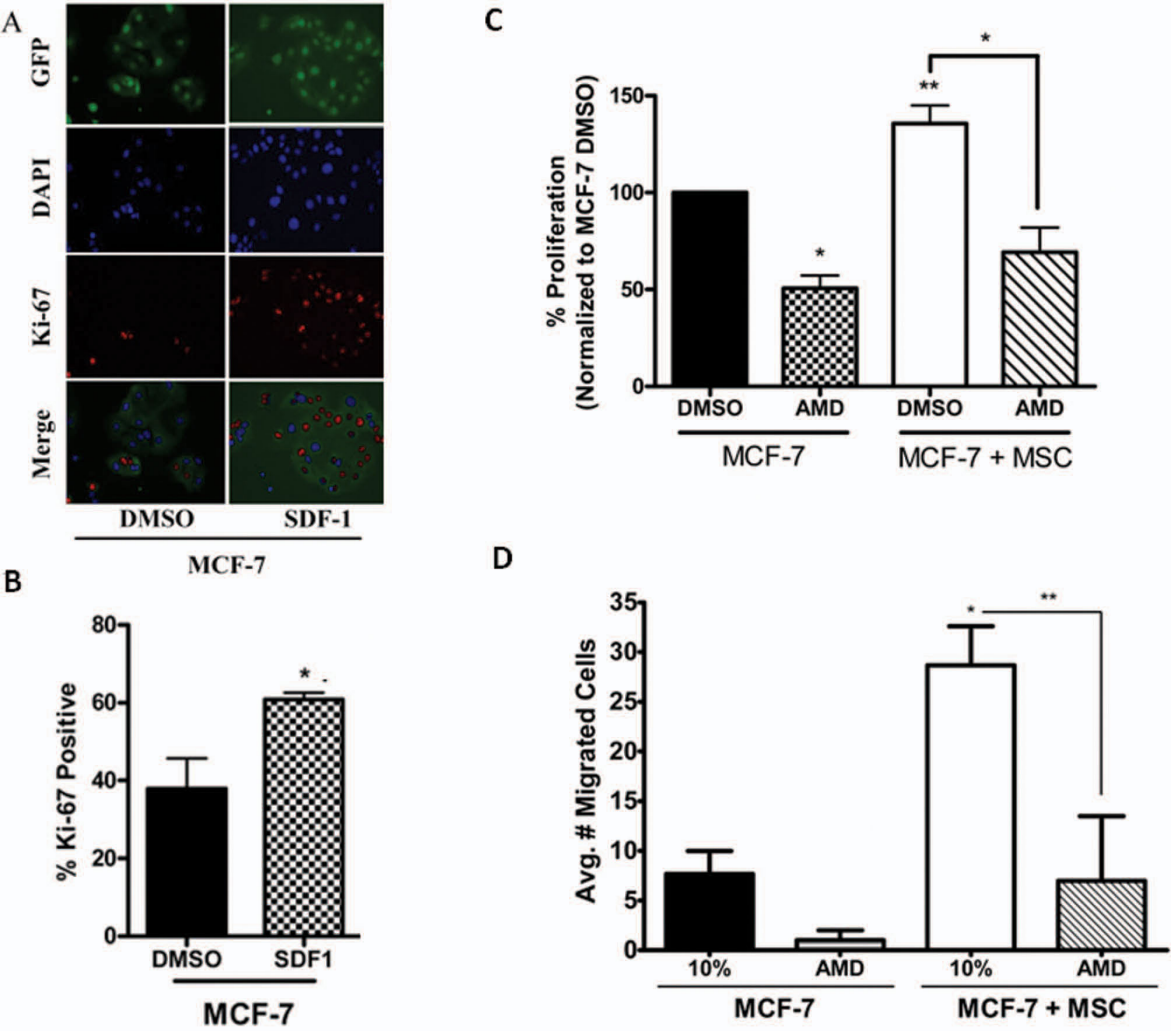
**Inhibition of CXCR4 signaling decreases hMSC stimulated MCF-7 proliferation and migration *in vitro***. (A) MCF-7 cells cultured alone were treated with DMSO (control) or SDF-1 (50 ng/ml) for 72 hours. Cells were fixed and stained with anti-Ki-67 (red) and nuclei were counter stained with DAPI (blue). Representative images of MCF-7 cells at 200×. (B) Quantification of cells positive for Ki-67 staining from 10 fields of view per treatment. Data is represented as percent positive cells of total cells counted. Bars represent mean values ± SEM. (C) MCF-7 cells were seeded in the lower chamber of a 24-well plate and a transwell insert was placed in each well. MCF-7 cells or hMSCs were seeded in the upper well. Upper and lower chambers were treated with DMSO (control) or AMD3100 (5 μg/ml). After 7 days of culture, inserts were removed and MTT was added to each well. After 4 hours cells were lysed and absorbance read. All data are represented as mean percent proliferation as compared to MCF-7 + DMSO (control) treated cells ± SEM. (D) MCF-7-GFP cells alone or in combination with hMSCs (1:1) were seeded in the upper chamber of a transwell system where the lower wells contained culture media supplemented with 10% FBS (10%). Upper and lower chambers were treated with DMSO (control) or AMD3100 (5 μg/ml). After 48 hours cells were fixed and the number of GFP-positive migrated cells counted. Bars represent average number of migrated cell per condition ± SEM, (*, p < 0.05; **, p < 0.01).

To determine if SDF-1 affects MCF-7 cell growth, MCF-7 cells were plated in phenol red-free reduced serum media and treated with SDF-1 or vehicle control. After 72 hours, cells were fixed and stained with Ki-67 as a marker of proliferation. MCF-7 cell proliferation increased with SDF-1 treatment 61% from 37.88 ± 7.84% positive to 60.85 ± 1.74% positive (Figure 5A-B, p < 0.05), demonstrating the proliferative effect of SDF-1 on MCF-7 cells.

Due to the increased SDF-1 gene expression observed *in vitro *and *in vivo *as well as the ability of SDF-1 to induce MCF-7 cell proliferation, we next examined CXCR4 signalling effects on hMSC-mediated proliferation. The small molecule inhibitor to CXCR4, AMD3100, was utilized in transwell proliferation assays to determine if blocking CXCR4 signalling is sufficient to ablate hMSC induced MCF-7 cell growth. Figure 5C demonstrates that inhibition of CXCR4 signalling decreases baseline proliferation in MCF-7 cells by 49.32% (from 100% to 50.68 ± 6.5%, p < 0.05). Furthermore, AMD3100 significantly decreased the effects of hMSC on MCF-7 proliferation by approximately 49% from 135.6 ± 9.37% to 69.24 ± 12.64% ki-67 positive (p < 0.05); however, AMD3100 treatment was unable to significantly inhibit proliferation compared to MCF-7 control levels. Similar to our results with ICI treatment, these data suggest that SDF-1/CXCR4 signalling is involved in the MCF-7/hMSC interaction, but inhibition is not sufficient to reverse the hMSCs effects. It is likely that multiple secreted factors and pathways play a role in communications between hMSCs and MCF-7 cells.

The role of CXCR4 signalling in the enhanced migratory response induced by hMSCs was also examined with AMD3100 treatment using the transwell migration assay. After 48 hours of culture with hMSCs, the number of migrated MCF-7 cells were counted and compared to MCF-7 cells cultured alone. AMD3100 treatment decreased the baseline migration of MCF-7 cells to a chemoattractant from 7.67 ± 2.33 cells to 1 ± 1 cell (Figure 5D), while AMD3100 treatment of co-culture cells resulted in a 4-fold decrease of MCF-7 cell migration (from 28.67 ± 3.93 cells to 7 ± 6.5 cells, p < 0.01). AMD3100 treatment was not able to inhibit migration below control levels, suggesting CXCR4 signalling is involved, but that it alone does not fully explain the hMSC induced effects on MCF-7 migration.

### Combined inhibition of ER and CXCR4 decreases hMSC mediated effects *in vitro*

**Figure 6:**
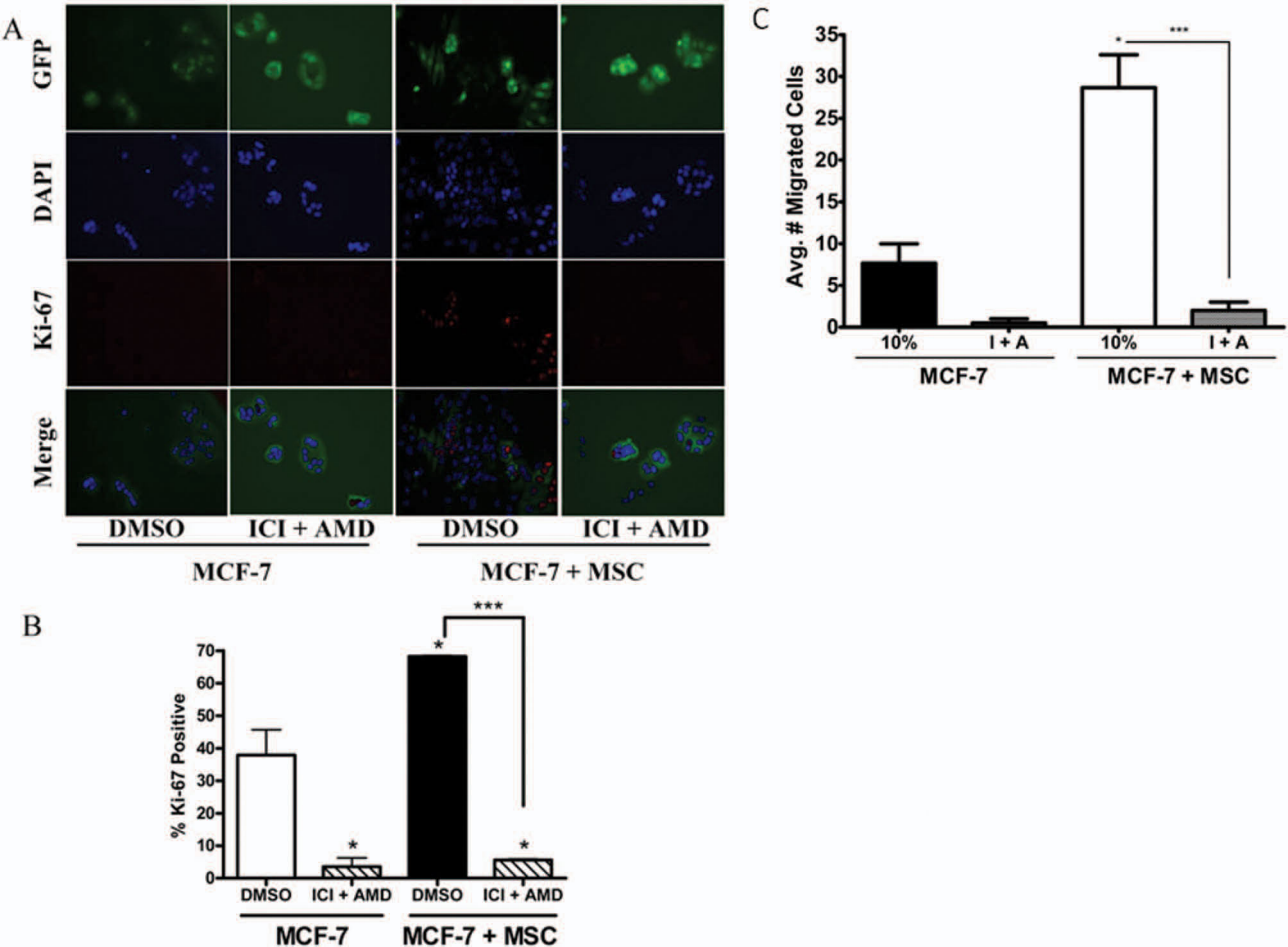
**Co-inhibition of ER and CXCR4 signaling decreases hMSC stimulated MCF-7 proliferation and migration *in vitro***. MCF-7-GFP cells cultured with or without hMSCs were treated with DMSO (control) or ICI (100 nM) + AMD3100 (5 μg/ml) for 72 hours. Cells were fixed and stained with anti-Ki-67 (red) and nuclei were counter stained with DAPI (blue). (A) Representative images of MCF-7 cells cultured alone (left) or with hMSCs (right) at 200×. (B) Quantification of GFP cells positive for Ki-67 staining from 10 fields of view per treatment. Data is represented as percent positive MCF-7 cells as compared to total number of MCF-7 cells counted. Bars represent mean values ± SEM. (C) MCF-7 cells alone or in combination with hMSCs (1:1 mix) were seeded in the upper chamber of a transwell system where the lower wells contained culture media supplemented with 10% FBS (10%). Upper and lower chambers were treated with either DMSO (control) or ICI (100 nM) + AMD3100 (5 μg/ml). After 48 hours cells were fixed and the number of migrated cells counted. Bars represent average number of migrated cell per condition ± SEM, (*, p < 0.05; ***, p < 0.001).

Both ER and CXCR4 signalling appear to be involved in the hMSC/MCF-7 cell interaction, yet inhibition of ER or CXCR4 alone does not sufficiently decrease hMSC induced effects. Thus, we next tested whether inhibition of both receptors simultaneously could reduce hMSC stimulated MCF-7 cell proliferation and migration. The effect of combined treatment with inhibitors ICI and AMD3100 was tested in direct co-culture immunofluorescent assays. Proliferation of MCF-7 cells was markedly decreased when treated simultaneously with ICI and AMD3100 regardless of hMSC presence or absence as determined by Ki-67 staining (Figure 6A). Quantification revealed that combined treatment reduced proliferation of MCF-7 cells cultured alone by 88% (from 37.88 ± 7.8% to 4.4 ± 3.6%, p < 0.05) and MCF-7 cells co-cultured with hMSCs by 92% ± 4.432% (from 68.3 ± 0.24% to 5.6 ± 0.4%, p < 0.001) (Figure 6B). Remarkably, combined inhibition of ER and CXCR4 was able to abrogate hMSC mediated effects on MCF-7 proliferation below control levels.

The effect of combined inhibition was also tested in the transwell migration assay. Figure 6C clearly indicates the addition of both ICI and AMD3100 resulted in significant decreases in the number of migrated cells from 7.67 ± 2.33 cells to 0.5 ± 0.5 cells under control conditions after 48 hours (p < 0.05). Moreover, inhibition of both ER and CXCR4 in MCF-7/hMSC co-culture conditions resulted in more than a 14 fold decrease in migration of MCF-7 cells (from 28.67 ± 3.93 cells to 2 ± 1, p < 0.001). Our results, in conjunction with recent reports, signify ER/CXCR4 crosstalk as a possible mechanism underlying the effects of hMSC on MCF-7 cell function [47,52].

## Discussion

Even though the therapeutic potential of hMSCs is an area of great excitement and promise, the possibility that hMSCs possess properties not conducive as vehicles for targeted cancer cell therapy exists [53,54]. Previous studies examining the effects of hMSCs on primary carcinoma cells have resulted in conflicting findings [45,53,55-57]. The variation of outcome from previous studies may be due to the source of hMSCs and donor variation, differences in culture and experimental techniques, the type and site of carcinoma, or a combination of these factors. Therefore, we set out here to determine the effect of hMSCs on the ER positive, hormone-dependent human breast carcinoma cell line MCF-7.

The effect of hMSCs on MCF-7 cell proliferation were tested under a variety of cell culture conditions including direct co-culture, indirect co-culture (transwell), and conditioned medium treatment. Proliferation assays revealed that hMSCs enhance MCF-7 cell proliferation *in vitro *under all conditions tested. The fact that transwell and conditioned medium experiments resulted in increased MCF-7 proliferation suggests that this effect is mediated by secreted factor(s). Direct co-culture resulted in the most dramatic proliferative response followed by transwell assays, while conditioned medium treatment resulted in the smallest observed change. These results indicate that while cell-to-cell contact is not required for hMSC-mediated proliferation of MCF-7 cells, cell communication/cell contact enhances the effect. This is most likely due to changes in the secretory profile of "activated" cells versus that of naïve cells [56]. Cells communicate with surrounding cells via direct contact and secreted factors and based on these signals, cells are induced to adapt in response to their environment. hMSCs are no exception; for instance, the proteins hMSCs secrete basally have been shown to be altered in the presence of UV irradiated fibroblasts as a response to tissue damage [58].

Karnoub *et al*. have demonstrated the ability of hMSCs to confer a metastatic phenotype to normally non/low metastatic cancer cells [49]. There have also been observations indicating that hMSCs decrease cell-to-cell contact and decrease epithelial cell adhesion markers (E-cadherin, ESA) of breast carcinoma cells [26,48,59]. Furthermore, we have previously demonstrated the ability of hMSCs to promote the progression of MCF-7 cells to hormone independence [45], a hallmark of primary tumour progression which signifies a more aggressive cancer phenotype [60]. In this study we set out to determine if hMSCs have the capability to affect the migration potential of MCF-7 cells, which have been characterized as having very low metastatic potential [61]. *In vitro *migration studies revealed an approximately 4-fold increase in migration of MCF-7 cells cultured in the presence of hMSCs under basal and stimulated chemotaxis conditions, suggesting the involvement of hMSCs in the promotion of a metastatic phenotype.

In addition to the ability of hMSCs to induce hormone-independent MCF-7 tumourigenesis, we have also demonstrated an increase in progesterone receptor expression in MCF-7-hMSC derived tumours indicative of enhanced oestrogen receptor signalling [45]. In this report we show that inhibition of ER decreases the hMSC-mediated enhanced proliferation of MCF-7 cells in the absence of exogenous oestrogen. Results from *in vitro *migration assays also revealed diminished hMSC-induced migration activity of MCF-7 cells grown when treated with ICI. Although the mechanism of hMSC activation of the ER is not completely understood at this time, our results establish a role for ER-mediated signalling in the hMSC-MCF-7 cell interaction.

The inability of ER inhibition to completely reverse the effect of hMSCs on MCF-7 cell proliferation and migration points to the existence of one or more additional pathways involved in the interaction between hMSCs and MCF-7 cells. hMSCs are known to secrete a number of factors, including growth factors and chemokines [26,48,49,62,63]. Secreted levels of SDF-1 by hMSCs have been shown to be altered based on microenvironmental factors [35,64]. Additionally, SDF-1 is an ER mediated gene recently implicated, with its receptor CXCR4, in crosstalk with ER to mediate hormone independence through activation of an autocrine feed-forward loop [47]. Examination of SDF-1 expression levels in our tumour samples revealed increased expression in hMSC + MCF-7 derived tumours both in the presence and absence of exogenous oestrogen, as well as *in vitro *samples.

Although the chemokine SDF-1 is mainly classified as a chemotactic factor mediating cell trafficking, it has also been shown to be involved in angiogenesis [65], survival [66] and cell proliferation [66-68]. Interestingly, treatment of MCF-7 cells with exogenous SDF-1 increased cell proliferation to practically equal levels of proliferation observed in cells co-cultured with hMSCs.

Since SDF-1 is an ER-regulated gene and appears to be involved in hMSC regulation of MCF-7 cell proliferation, the role of SDF-1 in MCF-7 cell proliferation and migration was examined in our model. Inhibition of CXCR4 signalling resulted in decreased proliferation of MCF-7 cells co-cultured with hMSCs, as well as decreased hMSC-enhanced migration of MCF-7 cells in response to a chemoattractant. These results demonstrate a role for SDF-1/CXCR4 signalling in the hMSC-MCF-7 cell interaction.

Similar to results from our ER inhibition studies, blocking SDF-1/CXCR4 signalling was not sufficient to completely reverse hMSCs effect on MCF-7 cell biology. Due to evidence of ER-CXCR4 crosstalk involvement in breast cancer progression, we examined the effects of simultaneous inhibition of ER and CXCR4 signalling to decrease hMSCs influence on MCF-7 cells. The effect of hMSC on proliferation and migration of MCF-7 cells was decreased to baseline control levels when both the ER and CXCR4 were inhibited, suggesting the involvement of ER-CXCR4 crosstalk in breast cancer progression.

The SDF-1/CXCR4 axis is clearly involved in hMSC-mediated effects on MCF-7 cell proliferation and migration; however it is not clear at this time the mechanism of SDF-1/CXCR4 activation in this system. Several possibilities exist: 1) hMSCs present at the tumour site respond to the tumour microenvironment by increased secretion of SDF-1. Secreted SDF-1 acts in a paracrine manor, binding CXCR4 present on MCF-7 cells, and stimulates ER in an oestrogen-independent fashion [47]. ER activation results in production of SDF-1 by the MCF-7 cell. SDF-1 secreted from MCF-7 cells can then bind CXCR4 on the cell surface completing the feed forward autocrine loop, or on neighbouring cells in a paracrine manor. 2) hMSCs may induce SDF-1 production in the MCF-7 cells through other secreted factors, or 3) hMSCs may activate ER signalling in turn inducing SDF-1 gene transcription. It is known that SDF-1/CXCR4 signalling can also function to increase cell proliferation through ER-independent mechanisms [69], which may explain why inhibition of the ER alone is not sufficient to completely abolish the hMSC effects on MCF-7 cells.

Targeting SDF-1/CXCR4 signalling has been proposed for the prevention and treatment of metastatic carcinoma, specifically of the breast [66,70-78]. Though the downstream mediators of this signalling are not completely clear, our research has implicated the involvement of both ER and CXCR4 signalling in hMSC driven hormone-independent tumourigenesis. The recent discovery of an ER/CXCR4 autocrine loop in breast carcinoma by Suave *et al*. supports our findings, though we are the first to suggest this as a mechanism driving hMSCs action on MCF-7 cell proliferation and metastasis [45,47].

## Conclusions

In this study we demonstrate the ability of hMSCs to promote proliferation and migration of the ER-positive, hormone-dependent breast cancer cell line MCF-7. Furthermore, we provide evidence for the involvement of ER and CXCR4 signalling in the hMSC-mediated effects on MCF-7 cell biology. These data provide insight into the complex relationship between tumour cells and the tumour microenvironment. In addition to our previous findings of hMSC-mediated hormone-independence of MCF-7 cells, the research presented here indicate the SDF-1/CXCR4 axis, in combination with ER-centric therapies, may also be a promising target for the treatment of hormone-independent and endocrine therapy-resistant, ER-positive breast carcinoma. Furthermore, we hope this research will encourage additional investigations into hMSC biological effects, ensuring proper precautions are taken for the use of hMSCs as a therapeutic tool.

## Methods

### Reagents

Dulbecco's modified Eagle's medium (DMEM), phenol-red free DMEM, fetal bovine serum (FBS), minimal essential amino acids (MEMAA), Non-essential amino acids (NEAA), antibiotic/anti-mitotic, penicillin/streptomycin (pen/strep), sodium pyruvate, L-glutamine, trypsin/EDTA, trypan blue stain (0.4%) and ethylenediaminetetraacetic acid (EDTA 0.5 M, pH8) were obtained from GIBCO (Invitrogen; Carlsbad, CA). Insulin, 17β-estrodiol (E2) and AMD3100 were purchased from Sigma-Aldrich (St. Louis, MO) and charcoal stripped (CS) FBS from HyClone (Thermo Scientific; Logan, UT). ICI 182,780 and SDF-1α were purchased from Tocris Bioscience (Ellisville, MO) and PeproTech, Inc (Rocky Hill, NJ), respectively. Alexa Fluor 555 Ki-67 immuno-fluorescence antibody and DAPI nuclear stain were purchased from BD Bioscience (San Jose, CA). Phosphate-Buffered Saline (PBS) was obtained from Cellgro (Mediatech, Inc.; Manassas, VA) and Dimethyl sulfoxide (DMSO) from Research Organics, Inc (Cleveland, OH).

### Cell Culture

MCF-7N cell variant is a subclone of the MCF-7 human breast adenocarcinoma cell line from the American Type Culture Collection (ATCC; Manassas, VA) that was generously provided by Louise Nutter (University of Minnesota, Minneapolis, MN) [79]. The MCF-7 human breast adenocarcinoma cell line was established more than 30 years ago from the pleural effusion of a patient with metastatic breast carcinoma [61]. The MCF-7 cell line retains characteristics of differentiated mammary epithelium and has been the model system of ER positive breast cancer since its discovery [80]. Prevalent use of MCF-7 cells has resulted in cell line variants reported to possess differing ER and PgR expression levels, oestrogen responsiveness, proliferation rates and TNF-α sensitivity [79,81-83]. The MCF-7 variant MCF-7N remains ER-positive, hormone dependent/oestrogen sensitive, and TNF-α and endocrine therapy sensitive making it a suitable model system for our studies. The MCF-7N variant line was used for all studies conducted in this publication. Cells were cultured as previously described [45,84]. Cells were maintained in Dulbecco's modified Eagle's medium (DMEM; pH 7.4; Invitrogen Corp., Carlsbad, CA) supplemented with 10% foetal bovine serum (Hyclone, Salt Lake City, UT), 1% NEAA, MEMAA, sodium pyruvate, antibiotic/anti-mitotic and insulin under mycoplasma-free conditions at 37°C in humidified 5% CO_2 _and 95% air. Human mesenchymal stem cells were maintained in 20% DMEM containing only 1% penicillin/streptomycin, sodium pyruvate and L-glutamine. In experiments requiring hormone or growth factor/cytokine treatment or when hormone independent effects were being assessed, phenol red-free DMEM supplemented with charcoal stripped FBS (10%), 1% NEAA, MEMAA, sodium pyruvate, penicillin/streptomycin, and L-glutamine was used (referred to as 10% CS media).

### Isolation and Culture of hMSCs

hMSCs from bone marrow aspirates were obtained from the Tulane University School of Medicine Adult Stem Cell Core and were prepared as described previously [85]. In brief, nucleated cells were isolated with a density gradient (Ficoll-Paque; Pharmacia) from 2-ml human bone marrow aspirated from the iliac crests of normal volunteers under a protocol approved by the Tulane University Institutional Review Board. All of the nucleated cells (30-70 million) were plated in a 145-cm^2 ^dish in 20-ml complete culture medium (CCM) that was prepared with 1 litre of alpha minimum essential media (α-MEM) (GIBCO, Rockville, MD), 200-ml FBS (lot-selected for rapid growth of MSCs; Atlanta Biologicals, Lawrenceville, GA), 100 units/ml penicillin, 100 μg/ml streptomycin, and 2 mM L-glutamine (GIBCO). After 24 hours at 37°C in 5% CO_2_, nonadherent cells were discarded, and incubation in fresh medium was continued for 4 days. The cells were lifted with 0.25% trypsin and 1 mM EDTA for 5 minutes at 37°C and then replated at 50 cells per cm^2 ^in an interconnecting system of culture flasks (6320 cm^2^; Nunc Cell Factory, Roskild, Denmark). Parallel 145-cm^2 ^dishes (Nunc) were plated under the same conditions as pilot samples to observe expansion of the cells. After cells in the pilot samples expanded 500- to 1,000-fold (7-9 days), the cells in the interconnecting flasks were lifted with trypsin/EDTA and frozen at a concentration of 1 × 10^6 ^cells/ml in liquid nitrogen as passage 1 cells. Alternatively, some samples were plated at high densities of 5,000 cells per cm^2 ^and incubated for 7-9 days prior to freezing. For most of the experiments here, a frozen vial of one million passage 1 cells was thawed, plated in 20 ml of CCM in a 145-cm^2 ^dish, and incubated for 1-3 days to recover viable passage 2 cells. The passage 2 cells were harvested with trypsin/EDTA and then incubated at 50-100 cells per cm^2 ^for 4-10 days and lifted with trypsin/EDTA to obtain passage 3 cells. During each step of expansion, the medium was changed every 3-5 days. Representative images from differentiation assays (Additional file 1, figure S1) and flow cytometry results for cell surface markers (Additional file 2, Table S1) used to define hMSCs are reported in additional files.

### Co-culture Assay

Co-culture methods were modified from Block *et al. *[58]. MCF-7-GFP cells were plated alone (2,000 cells per well) or in combination with hMSCs (1,000 cells each per well) in 200 μl phenol red-free DMEM with 10% CS FBS in a 96-well cell culture plate (Falcon; BD Bioscience; San Jose, CA). Cells were cultured for 72 hours under standard culture conditions and then subjected to IF staining (detailed below).

### Immuno Fluorescence Staining

After 72 hours of co-culture as outlined above, cells were fixed and stained for Ki-67 as modified from manufacturer's instructions and the publication by Kill *et al. *[86]. Briefly, cells were fixed using 100 μL of 3.7% formaldehyde in PBS for 10 minutes. Formaldehyde was removed and cells were permeabilized using cold (-20°C) 90% methanol for 5 minutes at room temperature and washed twice with PBS. 100 μL of blocking buffer (3% FBS in PBS) was then added. After 30 minutes, blocking buffer was removed and cells were incubated for 1 hour with Alexa Fluor-555 Ki-67 antibody (50 μL per well diluted 1:10 in blocking buffer; BD Pharmingen, San Diego, CA). Cells were then washed with PBS and stained with DAPI nuclear stain (1:1000) for 5 minutes before imaging. 5 fluorescence images per well (minimum of 10 images per treatment) were captured at 400×. Results are represented as percent positive Ki-67staining (red) of GFP positive MCF-7 cells (green). Nuclei are counter stained with DAPI (blue).

### Transwell Culture Assay

MCF-7 cells were plated at 10,000 cells per well in 1 ml of 20% DMEM in a 24-well plate. Transwell inserts (8 μm; BD biosciences; San Jose, CA) were placed into each well containing 5,000 cells, either MCF-7 (control) or hMSC, in 500 μl of appropriate culture media. Plates were cultured under normal conditions for 7 days, after which inserts were removed and cells subjected to MTT. For inhibitor studies 10% CS DMEM culture media was used and both upper and lower chambers were treated with ICI 182,780 (100 nM), AMD3100 (5 ug/ml) or both at the time of cell seeding. DMSO was used as vehicle control. Transwell culture protocol was modified from a previously published method [58].

### Generation of Conditioned Media

Conditioned media was generated based on methods previously published [36,87]. MCF-7 cells or hMSCs were plated to 70% confluency in T150 flasks (Corning; Corning, NY) in either 20% DMEM (proliferation) or 10% CS DMEM (qPCR) and allowed to adhere overnight at 37°C, 5% CO_2_. The next day media was removed and cells washed thrice with 1× sterile PBS. Cells were then re-fed with appropriate culture media. After 24 hours media was collected, spun down to remove cell debris (2,000 rpm × 5 minutes) and passed through 0.45 μm filter (Sigma-Aldrich; St. Louis, MO). CM aliquots were frozen at -20°C until needed (not exceeding 2 weeks).

### MTT Assay

*CM experiments*. Cells were plated at a density of 2.5 × 10^3 ^cells per well in a 96-well plate in 200 ul 20% DMEM and allowed to attach overnight. Cells were then treated with conditioned media from either MCF-7 cells or hMSCs for 24 hours. *Transwell experiments*. Cells were plated as described above. Following treatment, 20 μL (CM 96-well plate) or per well 200 μl (transwell 24-well plate) of MTT reagent (5 mg/ml) was incubated with cells for 4 hr. Media was aspirated and cells were lysed with 200 ul DMSO. 100 μl of cell lysates from transwell experiments were transferred to 96-well plates for absorbance readings. The absorbance was read on an ELx808 Microtek plate reader (Winooski, VT) at 550 nm, with a reference wavelength of 630 nm as previously described [88]. All experiments were conducted in triplicate. Data is represented as percent control proliferation ± SEM. Inhibitor studies (ICI 182,780 and AMD3100) were conducted simultaneously to more accurately compare the effects and therefore are reported using the same control values. The data has been split into multiple graphs to ensure clarity of interpretation and to demonstrate the effects on each pathway on cell proliferation.

### Transwell Migration Assay

Migration assays were performed based on the publication by Karnoub *et al*. and the manufacturer's instructions [49]. MCF-7-GFP cells were seeded either alone or in combination with hMSCs at a density of 2.5 × 10^4 ^(total cells; 1:1 mix) in 500 μl 10% CS DMEM in the upper chamber of a 24 well transwell system. Phenol red-free DMEM supplemented with 10% CS FBS (10%) was used as a chemoattractant in the lower wells. Phenol red-free DMEM without FBS (0%) was used as a negative control to assess basal migration rates. After 48 hours of treatment (DMSO, ICI 182,780, AMD3100, ICI + AMD), membranes were scrubbed to remove non-migrated cells and membranes were removed and mounted on glass slides. Migrated cells were visualized by microscopy and the number of GFP positive (MCF-7) cells counted. Data is represented as number of migrated cells per field of view ± SEM for triplicate experiments. Migration studies using inhibitors (ICI 182,780 and AMD3100) were conducted simultaneously to more accurately compare all treatment conditions. The results have been divided between several graphs to more clearly demonstrate the effects of each of the individual pathways on migration.

### RNA Extraction and cDNA synthesis

#### Tumour tissue RNA extraction

RNA was isolated from tumours extracted from mice at endpoint using Trizol LS (Invitrogen) with Purelink RNA purification system (Invitrogen) according to the manufacturer's protocol.

#### Conditioned Media qPCR

Cells were plated in 10 cm^2 ^dishes (Corning; Corning, NY) at 70% confluence and allowed to adhere overnight at 37°C, 5% CO_2_. The next day media was removed, cells washed thrice with 1× sterile PBS and CM (10 ml) from either MCF-7 (control) or hMSCs was added. Cells were harvested with PBS/EDTA after 24 hours of treatment. Total RNA was isolated from cell pellets using the RNeasy kit per manufacturer's instructions (Qiagen; Valencia, CA).

The quantity and quality of the RNA were determined by absorbance at 260 and 280 nm using the NanoDrop ND-1000 (NanoDrop; Wilmington, DE). Total RNA was reverse-transcribed using the iScript kit (BioRad; Hercules, CA).

### Quantitative Real Time RT-PCR

For qPCR forward and reverse primers were as follow: Actin: (F) 5'-TGA GCG CGG CTA CAG CTT-3', (R) 5' - CCT TAA TGT CAC ACA CGA TT - 3'; SDF-1: (F) 5' - AGT CAG GTG GTG GCT TAA CAG - 3', (R) 5' - AGA GGA GGT GAA GGC AGT GG - 3'; CXCR4: (F) 5' - AAA GTA CCA GTT TGC CAC GGC - 3', (R) 5' - GCA TGA CGG ACA AGT ACA GGC T - 3'. All primers were obtained from Invitrogen (Carlsbad, CA). The PCR reaction was carried out as follows: step 1: 95°C 3 minutes, step 2: for 40 cycles 95°C 20 seconds, 60°C 1 minute, step 3 70°C 10 seconds, hold at 4°C. Each reaction tube contained: 12.5 μl 2× SYBR Green supermix + 6.5 μl nuclease-free water + 1 μl 0.1 μg/μl primer (pair) + 5 μl cDNA (0.2 μg/μl). Genes were amplified in triplicate. Data was analyzed by comparing relative target gene expression to actin control. Relative gene expression was analyzed using 2^-ΔΔCt ^method [89]. RNA isolation, cDNA synthesis and qPCR were performed as previously described and outlined above [90-92].

### Microscopy

The Nikon eclipse TE2000-s inverted fluorescence microscope and camera with x-cite series 120 illuminator (Nikon; Melville, NY), in conjunction with IP Lab version 3.7 software (Rockville, MD) were used in the detection of IF staining. Fluorescence was observed under the following conditions (excitation/emission): Red - 555/565 nm; Blue - 358/461 nm; Green - 488/509 nm.

### Statistical Analysis

Studies involving more than 2 groups were analyzed by one-way ANOVA with Tukey's post-test using the Graph Pad Prism V.4 software program. All others were subjected to unpaired student's t-test. A value of p < 0.05 was considered statistically significant.

## Competing interests

The authors declare that they have no competing interests.

## Authors' contributions

LVR was involved in study design, performed the molecular and cellular in vitro and in vivo studies, carried out data acquisition and statistical analysis, drafted and submitted the manuscript. JWA was involved in MTT and Ki-67 assays. SEM participated in the in vivo studies. SE was involved in cell culture, particularly in regard to in vivo experiments, and manuscript revision. BSB provided project guidance and manuscript revision. MEB conceived the study, participated in its design and coordination, and provided manuscript revision. All authors read and approved the final manuscript.

## Supplementary Material

Additional file 1 **Figure S1 - Characterization of hMSC differentiation capacity**. Differentiation assays were performed on hMSC (donor 7032R) at passage 2 prior to use in all experiments reported here. Representative images of hMSCs under (A) control culture conditions, (B) osteogenic conditions (stained with Alzarin Red S for calcium), or (C) adipogenic conditions (stained with oil red O for lipid). Original magnification, 40×. Click here for file

Additional file 2 **Table S1 - Quantification of cell surface hMSC characterization markers by flow cytometry**. Flow cytometry results for various cell surface markers expressed as the % of gated cells that are positive in the total gated population. Click here for file

## References

[B1] Ali S Coombes RC Estrogen receptor alpha in human breast cancer: occurrence and significance J Mammary Gland Biol Neoplasia 2000 5 271 281 10.1023/A:1009594727358 14973389

[B2] Jordan VC Gottardis MM Robinson SP Friedl A Immune-deficient animals to study "hormone-dependent" breast and endometrial cancer J Steroid Biochem 1989 34 169 176 10.1016/0022-4731(89)90079-4 2626014

[B3] Brodie A Sabnis G Jelovac D Aromatase and breast cancer J Steroid Biochem Mol Biol 2006 102 97 102 10.1016/j.jsbmb.2006.09.002 17113978 PMC1868399

[B4] Jordan VC Long-term tamoxifen therapy to control or to prevent breast cancer: laboratory concept to clinical trials Prog Clin Biol Res 1988 262 105 123 3287387

[B5] Reed MJ The role of aromatase in breast tumors Breast Cancer Res Treat 1994 30 7 17 10.1007/BF00682737 7949206

[B6] Wakeling AE Bowler J ICI 182,780, a new antioestrogen with clinical potential J Steroid Biochem Mol Biol 1992 43 173 177 10.1016/0960-0760(92)90204-V 1525058

[B7] Clarke R Liu MC Bouker KB Gu Z Lee RY Zhu Y Skaar TC Gomez B O'Brien K Wang Y Hilakivi-Clarke LA Antiestrogen resistance in breast cancer and the role of estrogen receptor signaling Oncogene 2003 22 7316 7339 10.1038/sj.onc.1206937 14576841

[B8] Clarke R Skaar TC Bouker KB Davis N Lee YR Welch JN Leonessa F Molecular and pharmacological aspects of antiestrogen resistance J Steroid Biochem Mol Biol 2001 76 71 84 10.1016/S0960-0760(00)00193-X 11384865

[B9] Kuske B Naughton C Moore K Macleod KG Miller WR Clarke R Langdon SP Cameron DA Endocrine therapy resistance can be associated with high estrogen receptor alpha (ERalpha) expression and reduced ERalpha phosphorylation in breast cancer models Endocr Relat Cancer 2006 13 1121 1133 10.1677/erc.1.01257 17158758

[B10] Garcia M Derocq D Freiss G Rochefort H Activation of estrogen receptor transfected into a receptor-negative breast cancer cell line decreases the metastatic and invasive potential of the cells Proc Natl Acad Sci USA 1992 89 11538 11542 10.1073/pnas.89.23.11538 1454845 PMC50587

[B11] van Agthoven T Sieuwerts AM Meijer-van Gelder ME Look MP Smid M Veldscholte J Sleijfer S Foekens JA Dorssers LC Relevance of breast cancer antiestrogen resistance genes in human breast cancer progression and tamoxifen resistance J Clin Oncol 2009 27 542 549 10.1200/JCO.2008.17.1462 19075277

[B12] (EBCTCG) EBCTCG Effects of chemotherapy and hormonal therapy for early breast cancer on recurrence and 15-year survival: an overview of the randomised trials Lancet 2005 365 1687 1717 10.1016/S0140-6736(05)66544-0 15894097

[B13] Larionov AA Miller WR Challenges in defining predictive markers for response to endocrine therapy in breast cancer Future Oncol 2009 5 1415 1428 10.2217/fon.09.113 19903069

[B14] Musgrove EA Sutherland RL Biological determinants of endocrine resistance in breast cancer Nat Rev Cancer 2009 9 631 643 10.1038/nrc2713 19701242

[B15] Miller WR Larionov A Renshaw L Anderson TJ Walker JR Krause A Sing T Evans DB Dixon JM Gene expression profiles differentiating between breast cancers clinically responsive or resistant to letrozole J Clin Oncol 2009 27 1382 1387 10.1200/JCO.2008.16.8849 19224856

[B16] Bhowmick NA Neilson EG Moses HL Stromal fibroblasts in cancer initiation and progression Nature 2004 432 332 337 10.1038/nature03096 15549095 PMC3050735

[B17] Hanahan D Weinberg RA The hallmarks of cancer Cell 2000 100 57 70 10.1016/S0092-8674(00)81683-9 10647931

[B18] Orimo A Gupta PB Sgroi DC Arenzana-Seisdedos F Delaunay T Naeem R Carey VJ Richardson AL Weinberg RA Stromal fibroblasts present in invasive human breast carcinomas promote tumor growth and angiogenesis through elevated SDF-1/CXCL12 secretion Cell 2005 121 335 348 10.1016/j.cell.2005.02.034 15882617

[B19] Bissell MJ Radisky D Putting tumours in context Nat Rev Cancer 2001 1 46 54 10.1038/35094059 11900251 PMC2975572

[B20] Hu M Polyak K Molecular characterisation of the tumour microenvironment in breast cancer Eur J Cancer 2008 44 2760 2765 10.1016/j.ejca.2008.09.038 19026532 PMC2729518

[B21] Liotta LA Kohn EC The microenvironment of the tumour-host interface Nature 2001 411 375 379 10.1038/35077241 11357145

[B22] Hasebe T Sasaki S Sugitoh M Ono M Saitoh N Ochiai A Highly proliferative intratumoral fibroblasts and a high proliferative microvessel index are significant predictors of tumor metastasis in T3 ulcerative-type colorectal cancer Hum Pathol 2001 32 401 409 10.1053/hupa.2001.23915 11331957

[B23] Kuniyasu H Abbruzzese JL Cleary KR Fidler IJ Induction of ductal and stromal hyperplasia by basic fibroblast growth factor produced by human pancreatic carcinoma Int J Oncol 2001 19 681 685 11562741 10.3892/ijo.19.4.681

[B24] Dvorak HF Tumors: wounds that do not heal. Similarities between tumor stroma generation and wound healing N Engl J Med 1986 315 1650 1659 10.1056/NEJM198612253152606 3537791

[B25] Aghi M Cohen KS Klein RJ Scadden DT Chiocca EA Tumor stromal-derived factor-1 recruits vascular progenitors to mitotic neovasculature, where microenvironment influences their differentiated phenotypes Cancer Res 2006 66 9054 9064 10.1158/0008-5472.CAN-05-3759 16982747

[B26] Hombauer H Minguell JJ Selective interactions between epithelial tumour cells and bone marrow mesenchymal stem cells Br J Cancer 2000 82 1290 1296 10.1054/bjoc.1999.1093 10755403 PMC2374484

[B27] Ritter E Perry A Yu J Wang T Tang L Bieberich E Breast cancer cell-derived fibroblast growth factor 2 and vascular endothelial growth factor are chemoattractants for bone marrow stromal stem cells Ann Surg 2008 247 310 314 10.1097/SLA.0b013e31816401d5 18216538

[B28] Schichor C Birnbaum T Etminan N Schnell O Grau S Miebach S Aboody K Padovan C Straube A Tonn JC Goldbrunner R Vascular endothelial growth factor A contributes to glioma-induced migration of human marrow stromal cells (hMSC) Exp Neurol 2006 199 301 310 10.1016/j.expneurol.2005.11.027 16574102

[B29] Prockop DJ Marrow stromal cells as stem cells for nonhematopoietic tissues Science 1997 276 71 74 10.1126/science.276.5309.71 9082988

[B30] Haynesworth SE Baber MA Caplan AI Cell surface antigens on human marrow-derived mesenchymal cells are detected by monoclonal antibodies Bone 1992 13 69 80 10.1016/8756-3282(92)90363-2 1316137

[B31] Mackay AM Beck SC Murphy JM Barry FP Chichester CO Pittenger MF Chondrogenic differentiation of cultured human mesenchymal stem cells from marrow Tissue Eng 1998 4 415 428 10.1089/ten.1998.4.415 9916173

[B32] Pittenger MF Mackay AM Beck SC Jaiswal RK Douglas R Mosca JD Moorman MA Simonetti DW Craig S Marshak DR Multilineage potential of adult human mesenchymal stem cells Science 1999 284 143 147 10.1126/science.284.5411.143 10102814

[B33] Qiao C Xu W Zhu W Hu J Qian H Yin Q Jiang R Yan Y Mao F Yang H Human mesenchymal stem cells isolated from the umbilical cord Cell Biol Int 2008 32 8 15 10.1016/j.cellbi.2007.08.002 17904875

[B34] Paget S The Distribution of Secondary Growths in Cancer of the Breast The Lancet 1889 133 571 573 10.1016/S0140-6736(00)49915-0 2673568

[B35] Corcoran KE Trzaska KA Fernandes H Bryan M Taborga M Srinivas V Packman K Patel PS Rameshwar P Mesenchymal stem cells in early entry of breast cancer into bone marrow PLoS ONE 2008 3 e2563 10.1371/journal.pone.0002563 18575622 PMC2430536

[B36] Molloy AP Martin FT Dwyer RM Griffin TP Murphy M Barry FP O'Brien T Kerin MJ Mesenchymal stem cell secretion of chemokines during differentiation into osteoblasts, and their potential role in mediating interactions with breast cancer cells Int J Cancer 2009 124 326 332 10.1002/ijc.23939 19003962

[B37] Horwitz EM Prockop DJ Fitzpatrick LA Koo WW Gordon PL Neel M Sussman M Orchard P Marx JC Pyeritz RE Brenner MK Transplantability and therapeutic effects of bone marrow-derived mesenchymal cells in children with osteogenesis imperfecta Nat Med 1999 5 309 313 10.1038/6529 10086387

[B38] Kidd S Spaeth E Dembinski JL Dietrich M Watson K Klopp A Battula VL Weil M Andreeff M Marini FC Direct evidence of mesenchymal stem cell tropism for tumor and wounding microenvironments using in vivo bioluminescent imaging Stem Cells 2009 27 2614 2623 10.1002/stem.187 19650040 PMC4160730

[B39] Studeny M Marini FC Champlin RE Zompetta C Fidler IJ Andreeff M Bone marrow-derived mesenchymal stem cells as vehicles for interferon-beta delivery into tumors Cancer Res 2002 62 3603 3608 12097260

[B40] Djouad F Bony C Apparailly F Louis-Plence P Jorgensen C Noel D Earlier onset of syngeneic tumors in the presence of mesenchymal stem cells Transplantation 2006 82 1060 1066 10.1097/01.tp.0000236098.13804.0b 17060855

[B41] Haynesworth SE Baber MA Caplan AI Cytokine expression by human marrow-derived mesenchymal progenitor cells in vitro: effects of dexamethasone and IL-1 alpha J Cell Physiol 1996 166 585 592 10.1002/(SICI)1097-4652(199603)166:3<585::AID-JCP13>3.0.CO;2-6 8600162

[B42] Hall B Andreeff M Marini F The participation of mesenchymal stem cells in tumor stroma formation and their application as targeted-gene delivery vehicles Handb Exp Pharmacol 2007 263 283 full_text 17554513 10.1007/978-3-540-68976-8_12

[B43] Stich S Haag M Haupl T Sezer O Notter M Kaps C Sittinger M Ringe J Gene expression profiling of human mesenchymal stem cells chemotactically induced with CXCL12 Cell Tissue Res 2009 336 225 236 10.1007/s00441-009-0768-z 19296133

[B44] Orimo A Weinberg RA Stromal fibroblasts in cancer: a novel tumor-promoting cell type Cell Cycle 2006 5 1597 1601 10.4161/cc.5.15.3112 16880743

[B45] Rhodes LV Muir SE Elliott S Guillot LM Antoon JW Penfornis P Tilghman SL Salvo VA Fonseca JP Lacey MR Adult human mesenchymal stem cells enhance breast tumorigenesis and promote hormone independence Breast Cancer Res Treat 121 293 300 10.1007/s10549-009-0458-2 19597705 PMC4943323

[B46] Hall JM Korach KS Stromal cell-derived factor 1, a novel target of estrogen receptor action, mediates the mitogenic effects of estradiol in ovarian and breast cancer cells Mol Endocrinol 2003 17 792 803 10.1210/me.2002-0438 12586845

[B47] Sauve K Lepage J Sanchez M Heveker N Tremblay A Positive feedback activation of estrogen receptors by the CXCL12-CXCR4 pathway Cancer Res 2009 69 5793 5800 10.1158/0008-5472.CAN-08-4924 19584281

[B48] Fierro FA Sierralta WD Epunan MJ Minguell JJ Marrow-derived mesenchymal stem cells: role in epithelial tumor cell determination Clin Exp Metastasis 2004 21 313 319 10.1023/B:CLIN.0000046130.79363.33 15554387

[B49] Karnoub AE Dash AB Vo AP Sullivan A Brooks MW Bell GW Richardson AL Polyak K Tubo R Weinberg RA Mesenchymal stem cells within tumour stroma promote breast cancer metastasis Nature 2007 449 557 563 10.1038/nature06188 17914389

[B50] Rhodes LV Burow ME Human mesenchymal stem cells as mediators of breast carcinoma tumorigenesis and progression TheScientificWorldJOURNAL 2010 10 1084 1087 20563531 10.1100/tsw.2010.108 PMC5763693

[B51] Hwang JH Shim SS Seok OS Lee HY Woo SK Kim BH Song HR Lee JK Park YK Comparison of cytokine expression in mesenchymal stem cells from human placenta, cord blood, and bone marrow J Korean Med Sci 2009 24 547 554 10.3346/jkms.2009.24.4.547 19654931 PMC2719209

[B52] Sengupta S Schiff R Katzenellenbogen BS Post-transcriptional regulation of chemokine receptor CXCR4 by estrogen in HER2 overexpressing, estrogen receptor-positive breast cancer cells Breast Cancer Res Treat 2009 117 243 251 10.1007/s10549-008-0186-z 18807177 PMC2728144

[B53] Mishra PJ Mishra PJ Glod JW Banerjee D Mesenchymal stem cells: flip side of the coin Cancer Res 2009 69 1255 1258 10.1158/0008-5472.CAN-08-3562 19208837

[B54] Prockop DJ Olson SD Clinical trials with adult stem/progenitor cells for tissue repair: let's not overlook some essential precautions Blood 2007 109 3147 3151 10.1182/blood-2006-03-013433 17170129 PMC1852233

[B55] Sasser AK Mundy BL Smith KM Studebaker AW Axel AE Haidet AM Fernandez SA Hall BM Human bone marrow stromal cells enhance breast cancer cell growth rates in a cell line-dependent manner when evaluated in 3D tumor environments Cancer Lett 2007 254 255 264 10.1016/j.canlet.2007.03.012 17467167

[B56] Spaeth EL Dembinski JL Sasser AK Watson K Klopp A Hall B Andreeff M Marini F Mesenchymal stem cell transition to tumor-associated fibroblasts contributes to fibrovascular network expansion and tumor progression PLoS One 2009 4 e4992 10.1371/journal.pone.0004992 19352430 PMC2661372

[B57] Giordano A Galderisi U Marino IR From the laboratory bench to the patient's bedside: an update on clinical trials with mesenchymal stem cells J Cell Physiol 2007 211 27 35 10.1002/jcp.20959 17226788

[B58] Block GJ Ohkouchi S Fung F Frenkel J Gregory C Pochampally R Dimattia G Sullivan DE Prockop DJ Multipotent Stromal Cells (MSCs) are Activated to Reduce Apoptosis in Part by Upregulation and Secretion of Stanniocalcin-1 (STC-1) Stem Cells 2009 27 670 681 19267325 10.1002/stem.20080742 PMC4742302

[B59] Martin FT Dwyer RM Kelly J Khan S Murphy JM Curran C Miller N Hennessy E Dockery P Barry FP Potential role of mesenchymal stem cells (MSCs) in the breast tumour microenvironment: stimulation of epithelial to mesenchymal transition (EMT) Breast Cancer Res Treat 2010 124 317 326 10.1007/s10549-010-0734-1 20087650

[B60] Leonessa F Boulay V Wright A Thompson EW Brunner N Clarke R The biology of breast tumor progression. Acquisition of hormone independence and resistance to cytotoxic drugs Acta Oncol 1992 31 115 123 10.3109/02841869209088890 1622625

[B61] Soule HD Vazguez J Long A Albert S Brennan M A human cell line from a pleural effusion derived from a breast carcinoma J Natl Cancer Inst 1973 51 1409 1416 4357757 10.1093/jnci/51.5.1409

[B62] Fiedler J Leucht F Waltenberger J Dehio C Brenner RE VEGF-A and PlGF-1 stimulate chemotactic migration of human mesenchymal progenitor cells Biochem Biophys Res Commun 2005 334 561 568 10.1016/j.bbrc.2005.06.116 16005848

[B63] Muller A Homey B Soto H Ge N Catron D Buchanan ME McClanahan T Murphy E Yuan W Wagner SN Involvement of chemokine receptors in breast cancer metastasis Nature 2001 410 50 56 10.1038/35065016 11242036

[B64] Cristino S Piacentini A Manferdini C Codeluppi K Grassi F Facchini A Lisignoli G Expression of CXC chemokines and their receptors is modulated during chondrogenic differentiation of human mesenchymal stem cells grown in three-dimensional scaffold: evidence in native cartilage Tissue Eng Part A 2008 14 97 105 10.1089/ten.a.2007.0121 18333808

[B65] Schober A Chemokines in vascular dysfunction and remodeling Arterioscler Thromb Vasc Biol 2008 28 1950 1959 10.1161/ATVBAHA.107.161224 18818421

[B66] Dewan MZ Ahmed S Iwasaki Y Ohba K Toi M Yamamoto N Stromal cell-derived factor-1 and CXCR4 receptor interaction in tumor growth and metastasis of breast cancer Biomed Pharmacother 2006 60 273 276 10.1016/j.biopha.2006.06.004 16828253

[B67] Gelmini S Mangoni M Serio M Romagnani P Lazzeri E The critical role of SDF-1/CXCR4 axis in cancer and cancer stem cells metastasis J Endocrinol Invest 2008 31 809 819 18997494 10.1007/BF03349262

[B68] Smith MC Luker KE Garbow JR Prior JL Jackson E Piwnica-Worms D Luker GD CXCR4 regulates growth of both primary and metastatic breast cancer Cancer Res 2004 64 8604 8612 10.1158/0008-5472.CAN-04-1844 15574767

[B69] Akekawatchai C Holland JD Kochetkova M Wallace JC McColl SR Transactivation of CXCR4 by the insulin-like growth factor-1 receptor (IGF-1R) in human MDA-MB-231 breast cancer epithelial cells J Biol Chem 2005 280 39701 39708 10.1074/jbc.M509829200 16172123

[B70] Arya M Ahmed H Silhi N Williamson M Patel HR Clinical importance and therapeutic implications of the pivotal CXCL12-CXCR4 (chemokine ligand-receptor) interaction in cancer cell migration Tumour Biol 2007 28 123 131 10.1159/000102979 17510563

[B71] Du YF Shi Y Xing YF Zeng FQ Establishment of CXCR4-small interfering RNA retrovirus vector driven by human prostate-specific antigen promoter and its biological effects on prostate cancer in vitro and in vivo J Cancer Res Clin Oncol 2008 134 1255 1264 10.1007/s00432-008-0394-2 18431597 PMC12161737

[B72] Epstein RJ The CXCL12-CXCR4 chemotactic pathway as a target of adjuvant breast cancer therapies Nat Rev Cancer 2004 4 901 909 10.1038/nrc1473 15516962

[B73] He X Fang L Wang J Yi Y Zhang S Xie X Bryostatin-5 blocks stromal cell-derived factor-1 induced chemotaxis via desensitization and down-regulation of cell surface CXCR4 receptors Cancer Res 2008 68 8678 8686 10.1158/0008-5472.CAN-08-0294 18974109

[B74] Holm NT Abreo F Johnson LW Li BD Chu QD Elevated chemokine receptor CXCR4 expression in primary tumors following neoadjuvant chemotherapy predicts poor outcomes for patients with locally advanced breast cancer (LABC) Breast Cancer Res Treat 2009 113 293 299 10.1007/s10549-008-9921-8 18270814

[B75] Juarez J Bendall L Bradstock K Chemokines and their receptors as therapeutic targets: the role of the SDF-1/CXCR4 axis Curr Pharm Des 2004 10 1245 1259 10.2174/1381612043452640 15078139

[B76] Lapteva N Yang AG Sanders DE Strube RW Chen SY CXCR4 knockdown by small interfering RNA abrogates breast tumor growth in vivo Cancer Gene Ther 2005 12 84 89 10.1038/sj.cgt.7700770 15472715

[B77] Liang Z Wu H Reddy S Zhu A Wang S Blevins D Yoon Y Zhang Y Shim H Blockade of invasion and metastasis of breast cancer cells via targeting CXCR4 with an artificial microRNA Biochem Biophys Res Commun 2007 363 542 546 10.1016/j.bbrc.2007.09.007 17889832

[B78] Richert MM Vaidya KS Mills CN Wong D Korz W Hurst DR Welch DR Inhibition of CXCR4 by CTCE-9908 inhibits breast cancer metastasis to lung and bone Oncol Rep 2009 21 761 767 19212637 PMC13435714

[B79] Burow ME Weldon CB Tang Y Navar GL Krajewski S Reed JC Hammond TG Clejan S Beckman BS Differences in susceptibility to tumor necrosis factor alpha-induced apoptosis among MCF-7 breast cancer cell variants Cancer Res 1998 58 4940 4946 9810003

[B80] Levenson AS Jordan VC MCF-7: the first hormone-responsive breast cancer cell line Cancer Res 1997 57 3071 3078 9242427

[B81] Villalobos M Olea N Brotons JA Olea-Serrano MF Ruiz de Almodovar JM Pedraza V The E-screen assay: a comparison of different MCF7 cell stocks Environ Health Perspect 1995 103 844 850 10.2307/3432398 7498097 PMC1519213

[B82] Osborne CK Hobbs K Trent JM Biological differences among MCF-7 human breast cancer cell lines from different laboratories Breast Cancer Res Treat 1987 9 111 121 10.1007/BF01807363 3620713

[B83] Klotz DM Castles CG Fuqua SA Spriggs LL Hill SM Differential expression of wild-type and variant ER mRNAs by stocks of MCF-7 breast cancer cells may account for differences in estrogen responsiveness Biochem Biophys Res Commun 1995 210 609 615 10.1006/bbrc.1995.1702 7755640

[B84] Salvo VA Boue SM Fonseca JP Elliott S Corbitt C Collins-Burow BM Curiel TJ Srivastav SK Shih BY Carter-Wientjes C Antiestrogenic glyceollins suppress human breast and ovarian carcinoma tumorigenesis Clin Cancer Res 2006 12 7159 7164 10.1158/1078-0432.CCR-06-1426 17145841

[B85] Sekiya I Larson BL Smith JR Pochampally R Cui JG Prockop DJ Expansion of human adult stem cells from bone marrow stroma: conditions that maximize the yields of early progenitors and evaluate their quality Stem Cells 2002 20 530 541 10.1634/stemcells.20-6-530 12456961

[B86] Kill IR Localisation of the Ki-67 antigen within the nucleolus. Evidence for a fibrillarin-deficient region of the dense fibrillar component J Cell Sci 1996 109 1253 1263 8799815 10.1242/jcs.109.6.1253

[B87] Gnecchi M Melo LG Bone marrow-derived mesenchymal stem cells: isolation, expansion, characterization, viral transduction, and production of conditioned medium Methods Mol Biol 2009 482 281 294 full_text 19089363 10.1007/978-1-59745-060-7_18

[B88] Struckhoff AP Bittman R Burow ME Clejan S Elliott S Hammond T Tang Y Beckman BS Novel ceramide analogs as potential chemotherapeutic agents in breast cancer J Pharmacol Exp Ther 2004 309 523 532 10.1124/jpet.103.062760 14742741

[B89] Schmittgen TD Zakrajsek BA Mills AG Gorn V Singer MJ Reed MW Quantitative reverse transcription-polymerase chain reaction to study mRNA decay: comparison of endpoint and real-time methods Anal Biochem 2000 285 194 204 10.1006/abio.2000.4753 11017702

[B90] Tse C Capeau J [Real time PCR methodology for quantification of nucleic acids] Ann Biol Clin (Paris) 2003 61 279 293 12805005

[B91] Pfaffl MW A new mathematical model for relative quantification in real-time RT-PCR Nucleic Acids Res 2001 29 e45 10.1093/nar/29.9.e45 11328886 PMC55695

[B92] Zimmermann MC Tilghman SL Boue SM Salvo VA Elliott S Williams KY Skripnikova EV Ashe H Payton-Stewart F Vanhoy-Rhodes L Glyceollin I, a novel antiestrogenic phytoalexin isolated from activated soy J Pharmacol Exp Ther 2010 332 35 45 10.1124/jpet.109.160382 19797619 PMC2802480

