# Supplementary material for: Effects of human mesenchymal stem cells on ER-positive human breast carcinoma cells mediated through ER-SDF-1/CXCR4 crosstalk
Source: Mol Cancer. 2010 Nov 18;9:295. doi: 10.1186/1476-4598-9-295 (PMC2998478; doi:10.1186/1476-4598-9-295)
Supplement: Additional file 2 — Table S1 - Quantification of cell surface hMSC characterization markers by flow cytometry. Flow cytometry results for various cell surface markers expressed as the % of gated cells that are positive in the total gated population. [file 1476-4598-9-295-S2.DOC]

| **Panel No.** | **Antibody – Label** | **7032R P2**  **(% Gated)** |
| --- | --- | --- |
| 1 | CD36 - FITC | 0.23 |
|  | CD34 - PE | 0.14 |
|  | CD19 - ECD | 0.13 |
|  | CD11b - PeCy5 | 0.05 |
|  | CD45 - PeCy7 | 0.42 |
|  | | |
| 2 | PODXL - FITC | 23.58 |
|  | CD166 - PE | 99.64 |
|  | CD90 - PeCy5 | 99.45 |
|  | | |
| 3 | CD49b - FITC | 5.26 |
|  | CD105 - PE | 99.76 |
|  | CD117 - APC | 2.49 |
|  | CD3 - PeCy7 | 2.11 |
|  | | |
| 4 | CD147 - FITC | 92.15 |
|  | CD49c - PE | 99.70 |
|  | CD14 - ECD | 0.01 |
|  | CD29 - PeCy5 | 99.75 |
|  | | |
| 5 | CD59 - FITC | 99.73 |
|  | CD184 - PE | 6.76 |
|  | CD79a - PeCy5 | 0.19 |
|  | | |
| 6 | HLA-I:ABC - FITC | 91.37 |
|  | CD271 - PE | 1.23 |
|  | CD49f - PeCy5 | 31.71 |
|  | | |
| 7 | HLA-II:DR DQ DP - FITC | 8.31 |
|  | CD73a - PE | 99.02 |
|  | CD106 - PeCy5 | 3.17 |
|  | CD44 - APC | 99.18 |
|  | CD49d - PE | 18.32 |
|  | | |
| 8 | IgG1 FITC Isotype | 17.25 |
|  | IgG2a PE Isotype | 3.54 |
|  | IgG1 ECD Isotype | 1.11 |
|  | IgG1 PC5 Isotype | 0.06 |
